# Bayesian optimization for demographic inference

**DOI:** 10.1093/g3journal/jkad080

**Published:** 2023-04-18

**Authors:** Ekaterina Noskova, Viacheslav Borovitskiy

**Affiliations:** Computer Technologies Laboratory, ITMO University, St. Petersburg 197101, Russia; Department of Computer Science, ETH Zürich, Zürich 8092, Switzerland

**Keywords:** optimization, demographic history, Bayesian optimization, population genetics

## Abstract

Inference of demographic histories of species and populations is one of the central problems in population genetics. It is usually stated as an optimization problem: find a model’s parameters that maximize a certain log-likelihood. This log-likelihood is often expensive to evaluate in terms of time and hardware resources, critically more so for larger population counts. Although genetic algorithm-based solution has proven efficient for demographic inference in the past, it struggles to deal with log-likelihoods in the setting of more than three populations. Different tools are therefore needed to handle such scenarios. We introduce a new optimization pipeline for demographic inference with time consuming log-likelihood evaluations. It is based on Bayesian optimization, a prominent technique for optimizing expensive black box functions. Comparing to the existing widely used genetic algorithm solution, we demonstrate new pipeline’s superiority in the limited time budget setting with four and five populations, when using the log-likelihoods provided by the *moments* tool.

## Introduction

Population is a group of interbreeding individuals that are geographically close to each other. Over time, populations change their size; they split, merge, or sparingly interbreed with other populations. A record of such events is a *demographic history* of populations, an important subject of natural history. Various combinations of statistical models and optimization techniques enable us to uncover demographic histories of populations from sampled genetic data. Due to limited sample sizes, simplified nature of statistical models, and imperfections of optimization algorithms, the histories obtained this way are not the ultimate truth, but play an important role in verifying or guiding paleontological studies.

Reconstruction of a demographic history is called *demographic inference*. There exists a multitude of tools for demographic inference from *genetic data*, based on a variety of mathematical models (Gutenkunst *et al.* 2009; Excoffier *et al.* 2013, 2021; Jouganous *et al.* 2017; Steinrücken *et al.* 2019; Kamm *et al.* 2020; DeWitt *et al.* 2021). Each of these tools consists of two rather independent components: *likelihood computation* and *optimization*. The likelihood component evaluates *log-likelihood* of the observed data under a proposed demographic history. The optimization component takes in a *demographic model*—a parametric family of demographic histories—and searches for the parameters that maximize log-likelihood produced by the likelihood component.

Existing tools for demographic inference are usually limited in the number of analyzed populations. For example, the original version of the ∂a∂i tool by Gutenkunst *et al.* (2009) is able to handle only up to three populations. Another tool, *moments* by Jouganous *et al.* (2017), can handle only up to five populations. This is caused by computational complexity of likelihood evaluation techniques used therein, which would scale exponentially with the number of populations: for example, ∂a∂i numerically solves a partial differential equation whose dimension is equal to the number of populations. Some tools, e.g. *momi2* by Kamm *et al.* (2020), use methods that scale linearly with respect to the number of populations and are therefore able to handle an arbitrary number of them. This does not change the big picture though: different tools rely on different mathematical models with different assumptions and thus are not interchangeable.

In the end, demographic inference for multiple populations is widely regarded to be a slow and expensive procedure, making the problem of speeding it up to be a valuable research direction. One way to do this is to speed up the likelihood component of the tools. Along these lines, *moments* were presented as a faster but somewhat less accurate alternative to ∂a∂i as well as ∂a∂i itself received GPU support in Gutenkunst (2021) that allowed it to handle up to five populations. A whole other direction is to improve the optimization component. This was done in the first author’s previous work. It proposed a new tool *GADMA* (Noskova *et al.* 2020, 2022) that substitutes the optimization components of various tools—these are mostly based on (restarted) local search techniques—with a custom genetic algorithm, giving better performance.

However, even though some tools’ likelihood components can now handle up to five populations, e.g. ∂a∂i’s and *moments*’s, it is still recommended to use *GADMA* with up to three populations. This is a natural limitation stemming from genetic algorithm’s hunger for log-likelihood evaluations, something that becomes prohibiting for log-likelihoods whose evaluations are very expensive in terms of time and computational resources.

In this paper, we address this problem by introducing a new *Bayesian optimization*-based technique for demographic inference with larger numbers of populations and implementing it as part of the *GADMA* framework. Bayesian optimization (Shahriari *et al.* 2015) is a state of the art approach for optimizing expensive-to-evaluate functions within a limited (time) budget. From the data acquired by evaluating the target function, it *learns* a probabilistic model of this function and uses it to guide the choice of a new evaluation location. It comes with a cost though: Bayesian optimization’s inner workings are rather expensive, making it suitable only for problems where the target function is itself expensive to evaluate. Previous works used it for tuning large-scale systems (Snoek *et al.* 2012; Chen *et al.* 2018) or robot control policies (Berkenkamp *et al.* 2021; Jaquier *et al.* 2022) as well as for chemical reaction optimization (Shields *et al.* 2021), to name a few.

Our contribution is the following. We propose and implement a specialized Bayesian optimization pipeline for demographic inference with larger population counts. To choose this pipeline, we evaluate the performance of a number of candidates using *moments*’s likelihood component. In the same way, we then study the efficiency of the approach, expecting it to generalize to other expensive-to-evaluate settings. The results show rapid convergence of the proposed method on different datasets and prove its efficiency compared to the genetic algorithm in the settings of four and five populations

## Methods and materials

Here we introduce the methods we use, namely Bayesian optimization and Gaussian process regression, the machine learning technique Bayesian optimization relies upon, and also describe the datasets used further in the “Approach and results” section.

### Bayesian optimization

*Bayesian optimization* is a state of the art technique for optimizing expensive black box functions. It minimizes some *black box function*ϕ:T→R, i.e. a function we are able to evaluate at any input t∈T, obtaining a possibly noisy observation y(t) of ϕ(t), but nothing more (e.g. no gradients). Moreover, it is usually assumed that each such evaluation is expensive and the objective is to converge as fast as possible or to get as close to the global optimum as possible within a fixed budget of evaluations or time.

The main idea of Bayesian optimization is to use a relatively cheap surrogate model to approximate the expensive target function *ϕ* and use this model as a proxy to guide decisions. This procedure is performed on each optimization iteration anew, using the evaluations obtained thus far as data for training the model. The most widely used surrogate models are Gaussian processes (Rasmussen and Williams 2006) discussed in detail in “Gaussian process regression” section. The reason is their ability to perform well in small data regimes and to quantify uncertainty associated with their own predictions.

The optimization procedure begins by drawing a small random sample on the domain T and evaluating the target function at each of the drawn inputs. The obtained data are called the *initial design*. After this, a prior Gaussian process is chosen, usually from the options detailed later in the “Practical priors” section. This may be done manually, aided by some external considerations like prior knowledge about smoothness of the target function, or alternatively it may be chosen by means of the cross-validation procedure described in the “Cross-validation for prior selection” section.

At each optimization iteration, using as data the target function evaluations $t_{1},y_{1},\ldots ,t_{n},y_{n}$ obtained so far, Gaussian process regression is executed, as detailed in the “Gaussian process regression” section, resulting in a posterior Gaussian process $\hat{f}∼GP(\hat{m},\hat{k})$. The value $\hat{m}(t)=E\;\hat{f}(t)$ of its mean function $\hat{m}\;:\;T\to R$ at input *t* is treated as a *prediction* of the target function therein, while the value $\hat{k}(t,t)=\text{Var}\hat{f}(t)$ of its covariance function $\hat{k}\;:\;T\times T\to R$ at (t,t) is treated as the projected variance of this prediction, i.e. a *measure of uncertainty*.

Given the posterior Gaussian process, the location $t_{*}$ to evaluate the target function next is chosen by solving


$$t_{*}=\underset{t\in T}{\text{arg max}}\;\alpha (t),$$

where α:T→R is the *acquisition function* defined in terms of the posterior process $\hat{f}$ (Frazier 2018). Common choices of α include


(1) $$\alpha_{\mathrm{EI}}(t)=E\left(\max\left(0,\min_{1\leq i\leq I}y_{i}-\hat{f}(t)\right)\right),$$

(2) $$\alpha_{\mathrm{PI}}(t)=P\left(\hat{f}(t)<\min_{1\leq i\leq I}y_{i}\right),$$

(3) $$\alpha_{\mathrm{logEI}}(t)=E\left(\max\left(0,\min_{1\leq i\leq I}e^{y_{i}}-e^{\hat{f}(t)}\right)\right).$$

Here “EI” stands for expected improvement and “PI” stands for probability of improvement—which, respectively, is what these functions are meant to predict. The acquisition function $\alpha_{\mathrm{PI}}$ emphasizes high probability of getting *some* improvement over the current minimum, while the acquisition function $\alpha_{\mathrm{EI}}$ emphasizes getting the *maximal* possible improvement. The acquisition function $\alpha_{\mathrm{logEI}}(t)$ by Hutter *et al.* (2009) is the analog of $\alpha_{\mathrm{EI}}$ intended for use in conjunction with log-transformed data, i.e. when $y_{j}>0$ and $t_{1},\log\;y_{1},\ldots ,t_{n},\log\;y_{n}$ are fed into Gaussian process regression instead of $t_{i},y_{i},\ldots ,t_{n},y_{n}$. We will use it to model negative log-likelihood, which in many cases of interest is indeed positive. This means that for $\alpha_{\mathrm{logEI}}$ Gaussian process regression will be used to predict the logarithm of negative log-likelihood.

For a Gaussian process $\hat{f}$ with known $\hat{m}$ and $\hat{k}$, these acquisition functions may be computed in closed form (Hutter *et al.* 2009; Frazier 2018) and efficiently optimized by gradient descent (with restarts). Further we refer to the acquisition functions in Equations (1)–(3) by the names EI, PI, and LogEI, respectively.

**Fig. 1.**
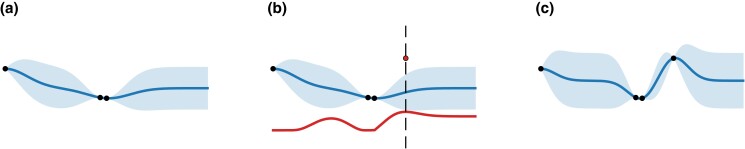
A fragment of Bayesian optimization’s workflow. The blue line (in the middle of the shaded region) is the prediction of the Gaussian process regression, i.e. $\hat{m}(\cdot )$, the shaded blue regions represent uncertainty bars whose height is proportional to $\hat{k}(\cdot ,\cdot {)}^{1/2}$. The red curve (line below) is the acquisition function. (a) Gaussian process regression is performed on previous target function evaluations (∙). (b) Function is evaluated (⚬) at the arg max (dashed line) of the acquisition function (red). (c) New data point is added, the regression is performed anew starting the next iteration.

A part of Bayesian optimization’s workflow is illustrated in Fig. 1.

### Gaussian process regression

Gaussian process regression models an unknown function *ϕ* from a dataset $t_{1},y_{1},\ldots ,t_{n},y_{n}$ where $y_{i}$ are (noisy) observations of $\phi (t_{i})$. It provides well-calibrated uncertainty bars alongside with its predictions. As its name suggests, it is based on *Gaussian processes*.

#### Gaussian processes

Mathematically, a *Gaussian process* is a family $\{f_{t}{\}}_{t\in T}$ of jointly Gaussian random variables $f_{t}$ indexed by some set T. Sometimes people reserve this term only for such index sets T that T⊆R but we will not stick to this convention. Instead, T will be a subset of $R^{d}$, where *d* is the number of parameters. Usually T is a product of segments corresponding to the domain of the modeled function *ϕ*.

A Gaussian process (or rather, strictly speaking, its distribution) is determined by a pair of deterministic functions: the *mean function*m:T→R and the *covariance kernel*k:T×T→R:


(4) $$m(t)=E(f_{t}),\;k(t,t^{\prime })=\text{Cov}(f_{t},f_{t^{\prime }}).$$

Conversely, each pair of deterministic functions *m* and *k* where *k* is positive semidefinite (see Rasmussen and Williams 2006 for the definition) constitutes a Gaussian process. This one-to-one correspondence motivates the standard notation


(5) f∼GP(m,k).

Because of their simplicity, Gaussian processes are attractive to encode distributions over functions. Furthermore, they turn out to be particularly suitable for the *Bayesian learning* framework that we briefly describe next.

#### Bayesian learning

The framework of *Bayesian learning* combines a *prior* Gaussian process f∼GP(m,k), some data $y\in R^{n}$ at locations $t\in T^{n}$, and a likelihood function p(y∣f) into the *posterior* process $\hat{f}$ given by the Bayes’ rule:


(6) $$p(\hat{f})\overset{\text{def}}{=}p(f|y)=\frac{\;p(y|f)p(f)}{p(y)}.$$

The posterior $\hat{f}$ is “similar” to the prior *f* but also respects the data y in a way prescribed by the likelihood p(y∣f). (Note that Equation (6) is non-rigorous: this form of Bayes’ rule treats the distributions of Gaussian processes as if they were absolutely continuous with respect to a finite dimensional Lebesgue measure, which they are not. The rigorous formalism exists of course, but we do not dwell on this here.)

The posterior process $\hat{f}$ may in general fail to be Gaussian, in which case any computations involving it will require complex numerical techniques like Markov chain Monte Carlo. Fortunately, if the likelihood function corresponds to the situation where for each observation $y_{i}$ one assumes $y_{i}=f(t_{i})+\varepsilon$ with $\varepsilon ∼N(0,\sigma_{\varepsilon }^{2})$ being Gaussian noise with variance $\sigma_{\varepsilon }^{2}$, then $\hat{f}$ is a Gaussian process. Moreover, $\hat{f}∼GP(\hat{m},\hat{k})$ with functions $\hat{m}$ and $\hat{k}$ given by the following simple closed form expressions (Rasmussen and Williams 2006), which we present, for further simplicity, under the assumption that m≡0.


(7) $$\hat{m}(\cdot )=K_{\cdot ,t}(K_{t,t}+\sigma_{\varepsilon }^{2}I{)}^{-1}y,$$

(8) $$\hat{k}(\cdot ,\cdot^{\prime })=k(\cdot ,\cdot^{\prime })-K_{\cdot ,t}(K_{t,t}+\sigma_{\varepsilon }^{2}I{)}^{-1}K_{t,\cdot^{\prime }},$$

where for a pair of vectors $a\in T^{l},b\in T^{s}$, the symbol $K_{a,b}$ denotes the l×s matrix defined by $\left(K_{a,b}{)}_{ij}=k(a_{i},b_{j}\right)$; $t=(t_{1},\ldots ,t_{n}{)}^{⊤}$ and similarly $y=(y_{1},\ldots ,y_{n}{)}^{⊤}$. As it was mentioned above, posterior mean $\hat{m}(t)$ represents a prediction at *t*. Note that it is a linear combination of observations $y_{1},\ldots ,y_{n}$ and its coefficients, given by the row-vector $K_{t,t}(K_{t,t}+\sigma_{\varepsilon }^{2}I{)}^{-1}$, are optimal in a certain sense (Stein 2012). The posterior variance $\hat{k}(t,t)$ represents a measure of uncertainty associated to the prediction at *t*: it is equal to the prior variance k(t,t) minus the term that quantifies information gained from observing y.

Because of this remarkable simplicity, the likelihood assumption corresponding to observations corrupted by Gaussian noise is often the setting of choice for applications. Importantly, it can be—and often is—used even when the observations are actually noiseless: in this case the noise can be interpreted as a model of the phenomena that cannot be captured by the Gaussian process.

Bayesian learning with Gaussian processes is the main part of the *Gaussian process regression* technique which we describe next.

#### Regression

As a preparatory step before *Gaussian process regression*, a parametric family of *priors*$f_{\theta }∼GP(m_{\theta },k_{\theta })$ must be selected. We postpone the discussion on specific families of priors used in practice until the “Practical priors”section, but right away make the widely used assumption of $m_{\theta }\equiv 0$, for simplicity. Note that this only means that the *prior* mean $m_{\theta }$ is zero, not the posterior mean $\hat{m}$. Moreover, in practice the observations are usually centered and scaled before feeding to Gaussian process regression, making zero mean a reasonable prior assumption.

After choosing such a family, the Gaussian process regression technique proceeds in two steps. First, the optimal parameters $\hat{\theta }$ of the prior and ${\hat{\sigma }}_{\varepsilon }^{2}$ of the likelihood function are found by maximizing the log marginal likelihood (Rasmussen and Williams 2006):


$$\begin{array}{rl} \hat{\theta },{\hat{\sigma }}_{\varepsilon }^{2} & =\underset{\theta ,\sigma_{\varepsilon }^{2}}{\text{arg max}}\;\log\;p_{\theta ,\sigma_{\varepsilon }^{2}}(y)\\ & =\underset{\theta ,\sigma_{\varepsilon }^{2}}{\text{arg max}}-\frac{1}{2}y^{⊤}(K_{\theta ,t,t}+\sigma_{\varepsilon }^{2}I{)}^{-1}y\\ & \;-\frac{1}{2}\log\;(\text{det}(K_{\theta ,t,t}+\sigma_{\varepsilon }^{2}I))-\frac{n}{2}\log\;(2\pi ). \end{array}$$

To solve this optimization problem, the simple gradient descent with multiple restarts is usually the tool of choice.

Step two of Gaussian process regression is to compute the Cholesky decomposition of the positive-definite matrix $K_{\hat{\theta },t,t}+{\hat{\sigma }}_{\varepsilon }^{2}I$, i.e. lay the groundword for solving linear systems of form


(9) $$(K_{\hat{\theta },t,t}+{\hat{\sigma }}_{\varepsilon }^{2}I)a=b.$$

After this is done, the prediction at an arbitrary location *t* is given by $\hat{m}(t)$ and the error bars therein are given by $\hat{k}(t,t)$.

The optimization problem which involves solving a linear system and computing a determinant, as well as the later step of computing the Cholesky decomposition have computational complexity $O(n^{3})$ where *n* is the size of the dataset. Searching for $\hat{\theta },{\hat{\sigma_{n}}}^{2}$ requires solving a linear system and computing a determinant at each iteration of the optimization procedure making it the super-cubic bottleneck in the computational complexity of the regression. This prohibits the use of this simple approach for large *n*. However, after the two steps are completed, computing predictions and error bars require only the relatively cheap—O(n) and $O(n^{2})$, respectively—and easily parallelizable matrix-vector products.

We now discuss practical Gaussian process priors.

#### Practical priors

In order to perform Gaussian process regression, a parametric family of priors $f_{\theta }∼GP(m_{\theta },k_{\theta })$ has to be selected. In practice, the prior mean $m_{\theta }$ is usually assumed to either be zero or an (optimizable) constant. With that said, we focus on choosing $k_{\theta }$ and assume $m_{\theta }\equiv 0$.

The most widely used family of prior Gaussian processes is the *Matérn family* (Rasmussen and Williams 2006; Stein 2012). It is parameterized by three positive parameters: (1) smoothness ν that determines how many derivatives the resulting Gaussian process will have, (2) length scale κ which scales the *t* axis, and (3) variance $\sigma^{2}$ which scales the *y* axis. Matérn family consists of the zero mean Gaussian processes with covariance kernels


(10) $$k_{\nu ,\kappa ,\sigma^{2}}(t,t^{\prime })=\sigma^{2}\frac{2^{1-\nu }}{\Gamma (\nu )}{\left(\sqrt{2\nu }\frac{\left\|t-t^{\prime }\right\|}{\kappa }\right)}^{\nu }K_{\nu }\left(\sqrt{2\nu }\frac{\left\|t-t^{\prime }\right\|}{\kappa }\right),$$

where $K_{\nu }$ is the Bessel function of the second kind and Γ is the gamma function (see definitions in Gradshteyn and Ryzhik 2014).

The general Matérn family given by Equation (10) is often divided into subfamilies corresponding to a single value of ν. Specifically, the cases of ν∈{1/2,3/2,5/2,∞} are usually considered, in which Equation (10) may be substantially simplified (Rasmussen and Williams 2006) to give


(11) $$k_{1/2,\kappa ,\sigma^{2}}(t,t^{\prime })=\sigma^{2}\exp\;\left(-\frac{u}{\kappa }\right),$$

(12) $$k_{3/2,\kappa ,\sigma^{2}}(t,t^{\prime })=\sigma^{2}\left(1+\frac{\sqrt{3}u}{\kappa }\right)\exp\;\left(-\frac{\sqrt{3}u}{\kappa }\right),$$

(13) $$k_{5/2,\kappa ,\sigma^{2}}(t,t^{\prime })=\sigma^{2}\left(1+\frac{\sqrt{5}u}{\kappa }+\frac{5u^{2}}{3\kappa^{2}}\right)\exp\;\left(-\frac{\sqrt{5}u}{\kappa }\right),$$

(14) $$k_{\infty ,\kappa ,\sigma^{2}}(t,t^{\prime })=\sigma^{2}\exp\;\left(-\frac{u^{2}}{2\kappa^{2}}\right)\;\mathrm{where}\;u=\|t-t^{\prime }\|.$$

The covariance given by $k_{1/2,\kappa ,\sigma^{2}}$ is also called the *exponential* kernel, the one given by $k_{\infty ,\kappa ,\sigma^{2}}$—the *Gaussian*, the *squared exponential*, or the *RBF* kernel. Subsequently, we refer to zero mean Gaussian processes with kernels (11), (12), (13), and (14) by Exponential, Matern32, Matern52, and RBF, respectively.

#### Cross-validation for prior selection

Although a parametric family of priors is often chosen manually, there are ways to automate this. Here we describe one popular way, termed *leave-one-out cross validation* (LOO-CV). It takes in some data, usually the initial design, and a collection of parametric families of priors. For each it computes a certain score, choosing the family with the highest.

Assuming a fixed parametric family of priors, the score is computed by leaving one datum $t_{i},y_{i}$ out of the data and computing the prediction quality *Q* at $t_{i}$ of the model ${\hat{f}}_{-i}$ obtained from the rest of the data, which we denote by $t_{-i},y_{-i}$. This is then averaged over all i∈{1,…,n}:


$$L_{\mathrm{LOO}-\mathrm{CV}}=\frac{1}{n}\sum_{i=1}^{n}Q(y_{i},{\hat{f}}_{-i}(t_{i})).$$

Assuming ${\hat{f}}_{-i}(t_{i})=N(\mu ,\sigma^{2})$, the prediction quality metric *Q* may be defined by


$$Q(y_{i},{\hat{f}}_{-i}(t_{i}))=-E(y_{i}-{\hat{f}}_{-i}(t_{i}){)}^{2}=-(y_{i}-\mu {)}^{2}-\sigma^{2}.$$

However, this metric clearly favors models that underestimate predictive uncertainty: a lower value of $\sigma^{2}$ directly causes *Q* to be higher. Because of this, another prediction quality metric is used, based on the likelihood, which is able to balance prediction quality with uncertainty calibration:


(15) $$Q(y_{i},{\hat{f}}_{-i}(t_{i}))=\log\;p_{{\hat{f}}_{-i}(t_{i})}(y_{i})$$

(16) $$=-\frac{(y_{i}-\mu {)}^{2}}{2\sigma^{2}}-\frac{1}{2}\log\;(2\pi \sigma^{2}).$$

Note that when the LogEI acquisition function is used, the value $y_{i}$ in Equation (16) must be substituted with $\log\;y_{i}$.

### Datasets

**Fig. 2.**
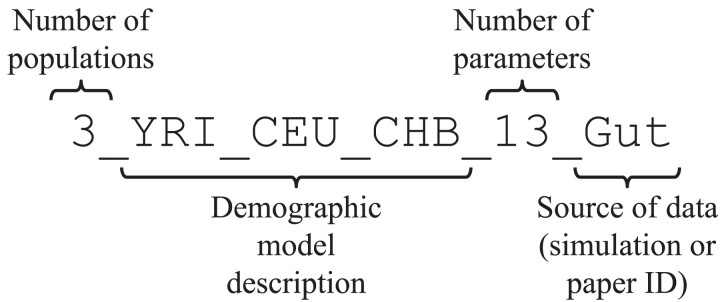
Dataset naming convention.

**Fig. 3.**
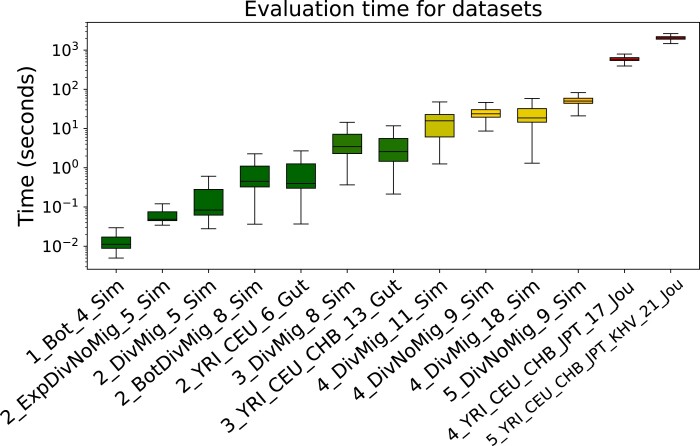
Evaluation times of log-likelihood by the *moments* tool for all used datasets. *Y*-axis is in log-scale. Boxplots are colored according to median value.

The log-likelihood functions provided by likelihood components of different tools are determined by the observed genetic data. To evaluate the performance of different optimization pipelines, we use several datasets from the package deminf_data that is available via https://github.com/noscode/demographic˙inference˙data. The genetic data in each of the datasets are represented by the allele frequency spectrum statistic, i.e. the histogram of the joint distribution of derived alleles of individuals. Additionally, each dataset contains a demographic model and bounds for the model’s parameters. Datasets are named according to the convention described in Fig. 2. More information about the datasets is available in the aforementioned repository deminf_data. The list of used datasets along with the corresponding times of log-likelihood evaluation is presented in Fig. 3.

Different Bayesian optimization pipelines studied in the “Approach and results” section are tested on the first 11 datasets. There is one dataset with one population, four datasets with two populations, two datasets with three populations, three datasets with four populations, and one dataset with five populations.

In the “Comparison to the genetic algorithm” section and the “Application to real data” section, additionally, two last datasets from Fig. 3 are used to (1) compare the final Bayesian optimization pipeline to the genetic algorithm implemented as part of the *GADMA* tool and (2) show that the final pipeline is able to find demographic models with higher log-likelihood values than reported in the literature. These datasets correspond to demographic models with four and five modern human populations and the genetic data built by Jouganous *et al.* (2017) from autosomal synonymous sequence data of the publicly available 1000 Genomes Project (Sudmant *et al.* 2015; The 1000 Genomes Project Consortium 2015). The data for four populations include: (1) Yoruba individuals from Ibadan, Nigeria (YRI); (2) Utah residents with northern and western European ancestry (CEU); (3) Han Chinese from Beijing, China (CHB); and (4) the Japanese from Tokyo (JPT). The data for five populations include the same four populations and (5) Kinh Vietnamese (KHV) population. These two datasets are also included in deminf_data package and are named 4_YRI_CEU_CHB_JPT_17_Jou for four populations and 5_YRI_CEU_CHB_JPT_KHV_21_Jou for five populations.

## Implementation

All Bayesian optimization pipelines discussed below were implemented as part of the open-source software *GADMA* available at https://github.com/ctlab/GADMA.

We highlight several of the ready-to-use libraries implementing Bayesian optimization: GPyOpt (The GPyOpt authors 2016), BOTorch (Balandat *et al.* 2020), and SMAC (Hutter *et al.* 2011; Lindauer *et al.* 2022). Unfortunately, GPyOpt is no longer supported since 2020. BOTorch is actively developed and popular, but SMAC proved itself well in a number of applications (Lago *et al.* 2018; Hewamalage *et al.* 2021; Wu *et al.* 2022) and, importantly, supports the LogEI acquisition function (Hutter *et al.* 2009) that will turn out to be a part of the best performing pipeline. Because of this, we use SMAC v0.13.1 to implement Bayesian optimization pipelines within *GADMA*.

## Approach and results

In this section, we propose and compare different candidate Bayesian optimization pipelines in the setting of demographic inference. After choosing the most fitting pipeline, we compare its performance to the genetic algorithm implemented as part of *GADMA* and show that it is able to attain unmatched log-likelihood values for a real-world dataset.

### Overview

To evaluate the performance of different candidate pipelines, we analyze convergence plots compiling 64 independent optimization runs using the datasets from the “Datasets” section and using the log-likelihoods provided by the *moments* tool. We use *moments* because, while being one of the most popular demographic inference tools alongside with ∂a∂i, it is not so time consuming as ∂a∂i, making it easier to do experimental evaluation. All experiments use the same hardware (Intel® Xeon® Gold 6248).

Candidate pipelines are determined by the choice of prior and acquisition function. We consider four zero mean Gaussian process priors with kernels from Equations (11)–(14) and three acquisition functions given by Equations (1)–(3), meaning that overall there are 12 candidates. We denote the candidates by Acquisition + Kernel, for example LogEI + Matern32. Initial design is always taken to be of size 2d where *d* is the number of demographic model’s parameters. It consists of pairs $t_{i},y_{i}$ with input locations $t_{i}$ sampled randomly from the same distribution that *GADMA* uses for its genetic algorithm implementation.

First, we try choosing the prior by comparing the leave-one-out cross-validation scores as it was described in the “Cross-validation for prior selection” section. We use large datasets (n=2,000) and evaluate the scores for 11 datasets from the “Datasets” section. The setup and results are detailed in the “Comparing cross-validation scores to choose a prior” section.

After this, we perform the most natural evaluation given the setting: we compare the candidate pipelines’ convergence performance on each of the datasets. This is detailed in the “Evaluating 12 basic candidates” section. We observe Matern52 and RBF kernels with PI and LogEI acquisition functions to show more or less equally good results, outperforming other candidates.

After this, we consider cross-validation scores on initial design as a tool for automatic prior selection, introducing additional candidate pipelines denoted by Acquisition + Auto. We evaluate performance of these for the two acquisition functions that performed best on the previous step. Both show equally good or better results compared to the four best performers of the previous step. The details may be found in the “Automatic prior selection” section.

Finally, we propose an ensemble pipeline denoted by Ensemble. On each optimization iteration, a coin is flipped. On heads the PI acquisition is used, on tails—LogEI. The prior is chosen by comparing the cross-validation scores, but in this case only between Matern52 and RBF which performed best in the previous experiments. This last candidate shows better or equal performance compared to the previous champions and is chosen as the final pipeline. See the detailed results in the “Ensembling” section.

To conclude, we compare the final pipeline Ensemble to *GADMA*’s genetic algorithm in terms of convergence speed as per iteration and as per wall-clock time, showing promising results that are presented in the “Comparison to the genetic algorithm” section. Immediately after, in the “Application to real data” section, we show that Ensemble can give better log-likelihood values than reported so far in the literature for a real data case study.

### Comparing cross-validation scores to choose a prior

One popular way of selecting the prior is by comparing the leave-one-out cross-validation scores (LOO-CV) as described by the “Cross-validation for prior selection” section. In an attempt to do so, we evaluated LOO-CV on 2,000 points in 11 datasets from the “Datasets” section and used those to compare four prior Gaussian processes under consideration. For each dataset, evaluation points were randomly sampled: the first 1,000 points were sampled from the uniform distribution on the target function’s prescribed domain, whereas the second 1,000 points were sampled from the non-uniform distribution *GADMA* uses to build initial design. Since the acquisition LogEI runs Gaussian process regression on the log-transformed data, we also evaluate the LOO-CV scores in this setting.

The results presented in Supplementary Tables S1 and S2 suggest that Exponential usually has worst performance and Matern52 is most often the best. However, there were outliers, for example the LOO-CV score for 4_DivMig_18_Sim dataset was best for Exponential. Overall, there is no single clear champion among the priors in terms of the LOO-CV score, however Matern52 comes closest.

### Evaluating 12 basic candidates

**Fig. 4.**
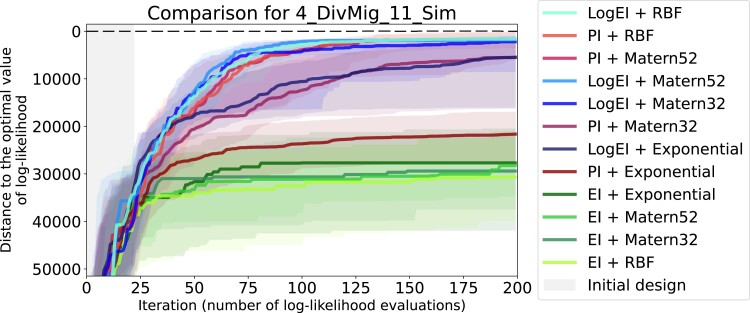
Convergence plot of 12 basic Bayesian optimization pipelines for the 4_DivMig_11_Sim dataset. For each candidate pipeline, 64 optimization runs were independently performed. Solid lines of different colors visualize the median of the sample of 64 values on each iteration, while shaded regions visualize ranges between the first and third quartiles. Gray area indicates the initial design, where random search is performed. The labels in the legend are sorted according to the final median.

The most comprehensive—although not very formal—way to evaluate the performance of different optimization pipelines is by examining the convergence plots, like the one given in Fig. 4. The somewhat informal criteria of comparison are: speed of convergence, interquartile distance and, most importantly, the end result. Convergence plots for all datasets with optimization ran for 200 iterations can be found in Supplementary Figs. S1–S3.

Candidates with Exponential prior or EI acquisition showed poor convergence for all of the 11 datasets. Matern32 usually showed equal or worse performance compared to Matern52 (see, e.g. Fig. 4). As the result, four candidates that combine Matern52 and RBF priors with PI and LogEI acquisitions were considered the most fitting to our setting.

We should note that results of examining the convergence plots are not very well aligned with the cross-validation scores discussed above. They agree in identifying Exponential as the worst choice, but disagree in some other cases. For example, in cases where LOO-CV scores suggest Exponential to be the best choice, convergence plots show the contrary. As another example, in Fig. 4 the PI + Matern32 candidate performs worse than PI + RBF candidate although Matern32 prior has better LOO-CV score than RBF prior (see Supplementary Table S1).

### Automatic prior selection

Because the results in the “Comparing cross-validation scores to choose a prior” section and the “Evaluating 12 basic candidates” section suggest that different priors fit different datasets, it is natural to consider automatic prior selection. We thus consider the following two new candidate pipelines. They select the prior that maximizes the LOO-CV score on the initial design data (all four possible priors are considered) and use one of the acquisition functions that performed best in previous experiments, namely PI and LogEI.

**Fig. 5.**
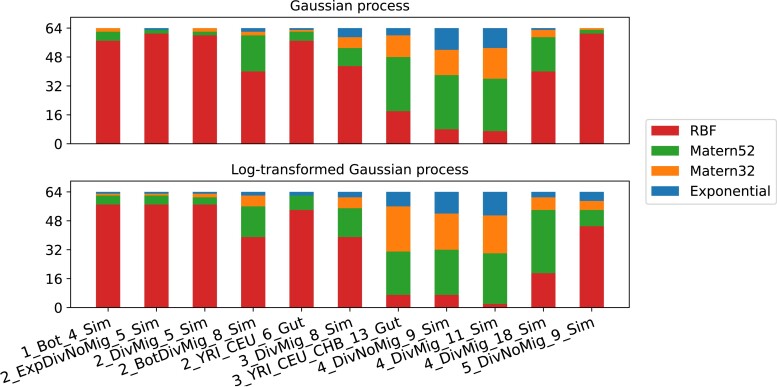
Histograms of automatic prior selection frequencies. The top row corresponds to the PI + Auto pipeline, while the bottom row corresponds to the LogEI + Auto pipeline.

We run these independently 64 times on 11 datasets from the “Datasets” section. The histograms of prior selection frequencies are presented in Fig. 5. The plots of convergence on 200 iterations are presented in Supplementary Figs. S4–S6. According to the histograms, frequency of prior selection varies for different datasets, however RBF and Matern52 are clearly the most frequent choices.

Upon examining convergence plots, it is clear that both new candidates show equal or better performance than the previous champions. For different datasets, especially corresponding to larger population counts, there was no clear winner. For instance, for three datasets 3_DivMig_8_Sim, 4_DivNoMig_9_Sim, and 4_DivMig_11_Sim, the pipeline LogEI + Auto shows better performance than PI + Auto. However, for two datasets 3_YRI_CEU_CHB_13_Gut and 5_DivNoMig_9_Sim, the results turned out the other way around.

### Ensembling

Ensembling different optimization techniques is a promising trick. By combining (e.g. by simply alternating) several methods, an ensemble is often able to give a considerably more efficient technique. For example, an ensemble Bayesian optimization technique named Squirrel (Awad *et al.* 2020) was one of the prize winners of the *Black box Optimization Challenge* in 2020.

Basing on these ideas, we propose yet another candidate pipeline. First, basing on the results of the “Comparing cross-validation scores to choose a prior” section and the “Evaluating 12 basic candidates” section, and on the frequencies of automatic prior choice examined in the “Automatic prior selection” section, we narrow down the set of priors to RBF and Matern52 and the set of acquisitions to PI and LogEI, deeming them the most efficient. The pipeline starts by choosing the best of the two priors based on the LOO-CV scores over the initial design. Then, on each iteration, one of the two acquisitions is chosen randomly (with equal probability). That is, on different iterations, different acquisition strategies are used. This pipeline is denoted by Ensemble.

**Fig. 6.**
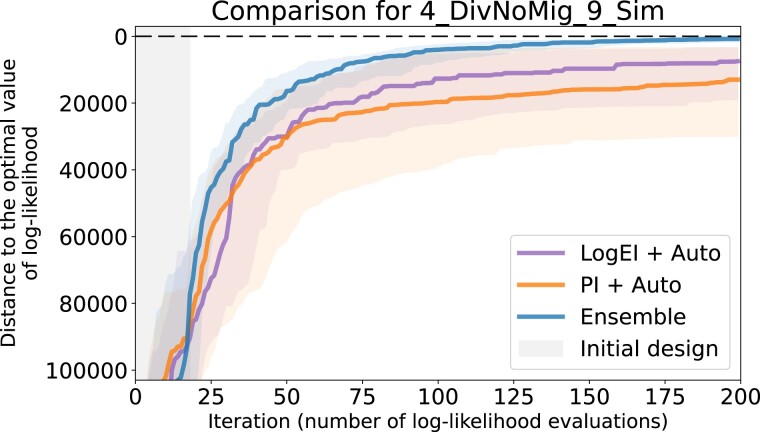
Convergence plots of the previously considered best performers and the new ensemble approach on the 4_DivNoMig_9_Sim dataset showing superiority of the latter. The meaning of the colored solid lines, shaded regions, and the gray area on the left are the same as in Fig. 4.

Comparing Ensemble to the previously mentioned best performers by examining convergence plots (Supplementary Figs. S7–S9) shows that it has similar or better performance. This makes the new Ensemble pipeline superior to all other candidates and naturally suggests choosing it as the final solution. It shows significant improvements on some datasets, e.g. on 4_DivNoMig_9_Sim (see Fig. 6) and 4_DivMig_11_Sim (see Supplementary Fig. S4).

### Comparison to the genetic algorithm

Finally, we compare the final champion pipeline Ensemble to the genetic algorithm implemented in second version of *GADMA* (Noskova *et al.* 2022). For this, besides the iteration-wise convergence plots used before, we use the wall-clock time convergence plots. These are important because, in contrast to the genetic algorithm, Bayesian optimization has tangible computational overhead arising from Gaussian process regression and acquisition function optimization ran on each iteration.

**Fig. 7.**
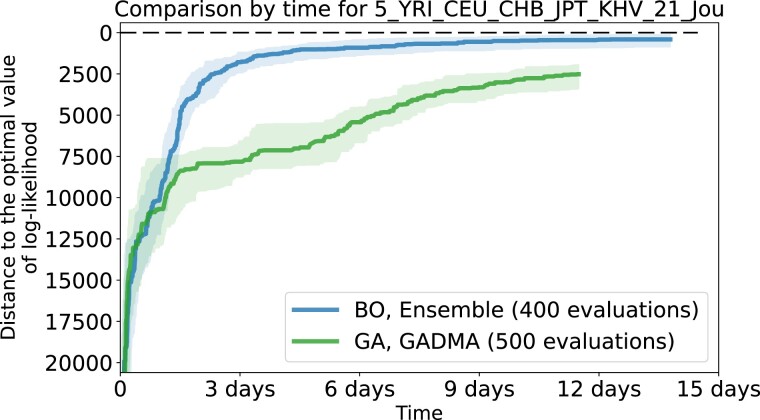
Wall-clock time convergence of Ensemble pipeline (blue, above) versus the genetic algorithm implementation from *GADMA* (green, below) on the 5_YRI_CEU_CHB_JPT_KHV_21_Jou dataset. The meaning of the colored solid lines and shaded regions are the same as in Fig. 4.

Two additional datasets of modern human populations from Jouganous *et al.* (2017) are included in this evaluation. The convergence plots for the total of 13 datasets are presented in Supplementary Figs. S10–S14. An example of a wall-clock time convergence plot is presented in Fig. 7.

The final Bayesian optimization pipeline turns out to be superior to the genetic algorithm on all datasets iteration-wise. However, as mentioned before, Bayesian optimization has overhead in terms of the wall-clock time, which makes the wall-clock time results different. Here, the more expensive-to-evaluate log-likelihood is, the better Bayesian optimization compares to the genetic algorithm. As demonstrated by Fig. 3, log-likelihood evaluation time depends on the dataset, mainly on the number of populations in this dataset.

In the end, the genetic algorithm turns out to be superior in terms of wall-clock time in cases of one and two populations. In the case of three populations, two optimization approaches show comparable results. In the case of four populations, Bayesian optimization has faster convergence in the beginning but then ties with the genetic algorithm. For five populations, however, Bayesian optimization turns out to be superior with a margin.

### Application to real data

Here, we pay closer attention to the additional datasets by Jouganous *et al.* (2017) that extend the standard out-of-Africa model by Gutenkunst *et al.* (2009) with three populations to the cases of four and five populations. These are exactly the dataset 4_YRI_CEU_CHB_JPT_17_Jou and the dataset 5_YRI_CEU_CHB_JPT_KHV_21_Jou, respectively. Jouganous *et al.* (2017) performed demographic inference for these datasets using the log-likelihoods provided by the *moments*’ tool and Powell’s method with restarts, reporting the resulting models and log-likelihood values. Demographic inference for these can bring real insights into the history of humankind and here we show that Ensemble is able to find new histories with higher log-likelihood values than ever previously reported.

The model for four populations has 17 parameters and the model for five has 21: the same 17 ones and four additional ones. To reduce computational time, Jouganous *et al.* (2017) optimized over 17 parameters of the former model, then fixed those and optimized over the remaining four parameters of the second model. We refer to the demographic histories obtained by Jouganous *et al.* (2017) as the *baseline histories*.

**Fig. 8.**
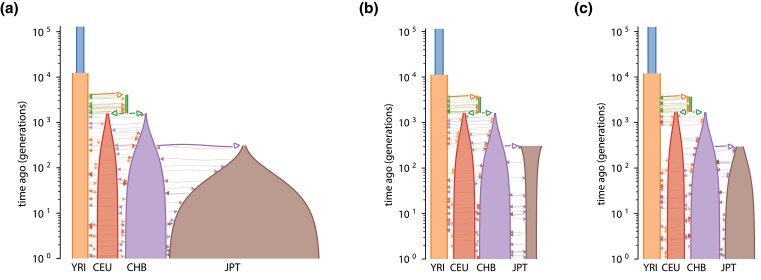
Visual representations of demographic histories of four human populations. Four human populations are presented: (1) Yoruba individuals from Ibadan, Nigeria (YRI); (2) Utah residents with northern and western European ancestry (CEU); (3) Han Chinese from Beijin, China (CHB); and (4) the Japanese from Tokyo (JPT). The histories (b) and (c) obtained using Bayesian optimization attain higher log-likelihood than the baseline history (a) from Jouganous *et al.* (2017). The figures (a–c) were generated using *demes* (Gower *et al.* 2022). a) Baseline history obtained using Powell’s method with restarts from Jouganous *et al.* (2017). b) Best history obtained using Bayesian optimization. c) Alternative history obtained using Bayesian optimization.

First, to infer a history (17 parameters) for the four populations model, we run Ensemble 64 times for 400 iterations followed by the BFGS (Broyden–Fletcher–Goldfarb–Shanno) local search until convergence. The results are presented in Supplementary Table S3 and Fig. 8. The best result has higher log-likelihood compared to the baseline history. The corresponding history is similar to the baseline but suggests exponential decline of JPT population from 30,000 to 15,000 individuals in contrast to the exponential growth from 4,000 up to 230,000 individuals in the baseline history (Fig. 8b). Moreover, the new history suggests a much lower migration rate between CHB and JPT populations. The second best history is much more similar to the baseline but has higher value of log-likelihood, lower growth rate of JPT, and lower migration rate between CHB and JPT populations (Fig. 8c).

After this, we fix the 17 parameters of the five populations model to the same values as in the baseline history, and run Ensemble for 200 iterations (followed by the local search) to infer the remaining four parameters. Interestingly, it requires only 16±7 iterations to overrun the baseline log-likelihood. The results are presented in Supplementary Table S4 and Fig. S15b. The history attaining best log-likelihood predicts migration rate between CHB and KHV populations to be twice as large as in the baseline history. Moreover, the split of CHB population that created KHV population is predicted earlier: 590 generations ago, as opposed to 337 generations in the baseline.

Finally, we use Ensemble to infer a demographic history for five populations from scratch, for the full 21 parameter model. We run Ensemble for 400 iterations followed by the local search, 64 independent times. The results are presented in Supplementary Table S4 and Fig. S15. The best result achieves higher log-likelihood value than both the baseline history and the history inferred by Ensemble with 17 parameters fixed (Supplementary Fig. S15c). It suggests exponential decline of JPT population, similar to the best history for four populations. This result is, however, hardly supported by other studies: the population divergence times are estimated to be very early, implying the out-of-Africa event more than a million years ago. The second best model is much better aligned with the contemporary knowledge (Supplementary Fig. S15d). The differences in parameters between this model and the baseline model concern mainly YRI and CEU populations. It could be explained by the low number of samples from these two populations in the dataset where allele frequency spectrum was downsized to five chromosomes for YRI and CEU populations and down to 30 chromosomes for the other three populations.

## Discussion

We proposed a Bayesian optimization pipeline suitable for demographic inference with expensive log-likelihood evaluations and limited resources. This pipeline was chosen as the single best performer in a series of experiments comparing different Bayesian optimization pipelines. In terms of the number of log-likelihood evaluations before attaining a good result, it was superior to *GADMA*’s genetic algorithm on all datasets. In terms of the wall-clock time, it was superior for optimizing the expensive-to-evaluate log-likelihoods in cases of four and five populations, showing that it can save days and weeks of computations.

Bayesian optimization was able to find demographic histories attaining higher log-likelihood than previously reported in Jouganous *et al.* (2017) for four and five modern human populations. However, the histories with the highest log-likelihood are not supported by current understanding of human history and may be anthropologically implausible. We highlight two possible reasons. First, even though Bayesian optimization performed better than the local search used in the original paper, given the high-dimensional nature of the optimization problem (21 parameters), it is quite probable that it did not find the global optimum. Second, it is known that allele frequencies may fail to uniquely determine demographic histories. For instance, it was shown that spectrum of a panmictic population can be a result of several different demographic scenarios (Rosen *et al.* 2018). Thus, it might be important to explore different local optima. Notably, in the case of four populations, the history attaining the second highest log-likelihood is close to the history from Jouganous *et al.* (2017), still surpasses the reported log-likelihood and is anthropologically plausible.

The following two paragraphs suggest practical considerations for using the proposed approach, based on its properties and empirical observations.

It is widely presumed that fewer parameters to be optimized make Bayesian optimization more efficient, with 20 being the critical value after which Bayesian optimization is likely to fail (Frazier 2018). Although in our experiments with up to 21 parameters Bayesian optimization performed well, the setting where 17 parameters were fixed and then only four optimized is noteworthy. There, it took only around 20 log-likelihood evaluations to overrun the best log-likelihood reported in the literature, which is an impressive result. This suggests that iteratively optimizing nested models whenever appropriate might be quite beneficial. We should also note that adjusting the final result of Bayesian optimization by a local search algorithm might be quite important to make the most out of it.

Bayesian optimization is best suited in scenarios with a priori fixed budget, like time and number of computation cores. In our implementation, a user should manually specify the number of iterations (equal to the number of log-likelihood evaluations) that Bayesian optimization should undertake. As noted above, Bayesian optimization could converge quite fast, especially when the parameter count is low. Moreover, because of Bayesian optimization’s own computational overhead (that increases with time), it is not recommended to run it for too many iterations, even if the target function is not expensive. We suggest to keep the number of iterations under 1,000, with 200 or 400 being good practical choices.

We note that for up to three populations, *GADMA* allows model free demographic inference by considering more flexible demographic structures (Noskova *et al.* 2020) instead of models. This requires solving optimization problems that involve discrete parameters. Bayesian optimization can be extended to handle these (in fact, the SMAC engine already has this feature) which may in future allow model free demographic inference for four and more populations, if an appropriate alternative to demographic structures is proposed and implemented.

## Supplementary Material

jkad080_Supplementary_Data

## Data Availability

The proposed Bayesian optimization for demographic inference is available as part of the open-software GADMA available via https://github.com/ctlab/GADMA. The datasets used are stored in the deminf_data package that is available at https://github.com/noscode/demographic˙inference˙data. Supplementary material is available at G3 online.
